# Factors affecting quality of life in women with breast cancer: a path analysis

**DOI:** 10.1186/s12905-023-02755-9

**Published:** 2023-11-08

**Authors:** Farnaz Faroughi, Azita Fathnezhad-Kazemi, Parvin Sarbakhsh

**Affiliations:** 1 Department of Midwifery, Faculty of Nursing and Midwifery, Maragheh Branch, Islamic Azad University, Maragheh, Iran; 2 Department of Midwifery, Women’s Reproductive and Mental Health Research Center, Tabriz Medical Sciences, Islamic Azad University, Tabriz, Iran; 3 Department of Midwifery, Faculty of Nursing and Midwifery, Tabriz Medical Sciences, Islamic Azad University, Tabriz, Iran; 4 Department of Statistics and Epidemiology, School of Public Health, Tabriz University of Medical Sciences, Tabriz, Iran

**Keywords:** Resilience, Quality of life, Hope, Support, Breast cancer

## Abstract

**Background:**

Breast cancer may negatively affect people’s quality of life. We investigated the predictors of quality of life in women with breast cancer with the mediating role of resilience.

**Method:**

In a cross-sectional design, 218 patients completed a survey referring to the Valiasr International Hospital Oncology Center in Tabriz, Iran. Four validated self-report measures assessed HRQoL as measured by the SF-12, Resilience, Hope, and Perceived Social Support (MSPs). The mediating roles of resilience between HRQoL and the fitness of the proposed model were investigated using path analysis. SPSS version 24 software and Lisrel 8.8 software were used for data analysis.

**Results:**

The results of path analysis showed that the final model had a good fit to the data (Chi-Square/ degrees of freedom (Normed Chi2) = 2.08, RMSEA = 0.014, goodness fit index = 0.99, both comparative fit index = 0.99 both CFI = 0.99 and IFI = 1). In this model, age and psychosocial factors predicted health-related quality of life.

**Conclusions:**

Age and psychosocial factors especially social support are important components in predicting health-related quality of life among those suffering from breast cancer.

## Background

Cancer, one of the most dangerous and complicated diseases, is associated with various factors such as environmental, genetic, social, cultural, ethnic, geographical, and many other unknown factors causing irreversible damage [1, 2]. Cancer has been predicted to become the most significant and leading cause of human death by 2030 [3]. Breast cancer is considered the most common neoplasm in women, and its resultant mortality rate is increasing every year [2, 4]. Statistics show that each year there are over 1.1 million newly diagnosed women with breast cancer worldwide and 410,000 women die from the disease [5]. Breast cancer constitutes 25% of all cancer cases in women In Iran [6]. Some diseases, particularly cancer development, lead to reduced quality of life of afflicted patients due to their mental and physical effects [7–9]. Research has shown that the diagnosis and treatment of cancers trigger a variety of negative emotional changes, such as stress, anxiety, fear, and significant depression [10–12], and these emotional responses can considerably disrupt the quality of life [8, 12, 13]. Quality of life is a crucial factor in evaluating the treatment effects and patients’ functional capabilities throughout their lives [14]. Researchers report that cancer patients with similar diseases and treatment conditions possess different levels of quality of life, which may be due to the effects of various factors, particularly psychosocial factors [15], such as perceived social support status, resilience, and hope [8, 16]. The mentioned factors reflect an individual’s ability to cope with the created conditions [17]. Biological factors (gene-environment), personal factors (e.g., sense of coherence, optimism, and hope), and most importantly, social factors (e.g., social support) are influential in cancer patient’s resistance and, consequently, continuation of the favorable treatment [18] so that the social environment is a crucial determinant that can impact the cancer patient’s ability to cope with stressful factors [16, 19]. Social support systems are among the essential protective factors for individuals who can control stressful events [20]. Furthermore, resilience is an individual’s ability to deal with significant changes, problems, and risks successfully [20, 21]. Evidence shows that resilience can play a critical role in maintaining the mental health of vulnerable populations suffering from stressful events, particularly in diseases with a high psychological burden, is considered a defense mechanism to cope with cancer and treatment-related problems [22], and is a significant protective factor against distress, so it is closely related to mental health. Research indicates that resilience positively affects the quality of life of afflicted patients [16, 23]. Besides the two factors of resilience and social support, hope can also play a remarkable role in promoting the quality of life [24]. Research has shown that there is a noteworthy connection between health, hope, and life satisfaction in cancer patients. Hope is a crucial aspect of a patient’s personality during times of distress, uncertainty, and discomfort. Hence, examining the associations between resilience, social support, and quality of life in individuals with bladder cancer has revealed a positive correlation between these factors [25]. Moreover, researchers have reported that resilience has a mediating effect between social support and quality of life in patients with multiple sclerosis and the elderly [26, 27]. Another issue is that having a hope, and receiving enough social support enhance resilience over time in a patient diagnosed with cancer [23, 28]. Based on the researchers’ report, resilience positively affects social participation in individuals’ lives by affecting hope [23].

On the other hand, numerous studies have shown that specific sociodemographic and patient characteristics exert varying impacts on social functioning, mental well-being, and overall quality of life for women diagnosed with breast cancer [15, 29]. Some researchers have reported adolescents and young adults with cancer have markedly poorer outcomes throughout their cancer journey, compared to children and older adults [30, 31]. It has been well-documented that young adults face some challenges including distress, fear of recurrence, spiritual, and existential despair, social isolation, problems with family communication and relationships, and disruptions in intimacy and independence [32]. Based on the findings of various studies, there appears to be a notable correlation between age and levels of resilience [33]. It is worth noting that different studies have presented conflicting perspectives, with some reporting a negative relationship and others indicating a positive one [34]. However, there is a paucity of research examining the association between age and resilience among adults with cancer. In addition, researchers have investigated the effect of various factors on hope, for example, Ballard et al. [35] compared the level of hope in newly diagnosed cancer patients to that of patients experiencing a cancer relapse. No difference was found in the Hope scores between the two groups. Studies show that middle-aged patients vs. older cancer patients vary in their way of coping with the illness, and one study noted that the total hope score had an inverse relationship to age [36].

Because of progress in early diagnosis and treatment, the survival rate of cancer patients has increased relative to the past, and research shows that health-related quality of life can positively affect the length of survival [37]. Considering the importance of the category of quality of life and identifying the factors contributing to it, and given the prevalence of breast cancer and the importance of its effect on all life aspects of afflicted individuals, the current research aims to investigate the factors affecting the quality of life of women with breast cancer.

Despite previous studies in this field, we embarked on a new research endeavor to investigate the effects of various factors in a comprehensive model and to examine both the direct and indirect impacts of these factors.

**Fig. 1:**
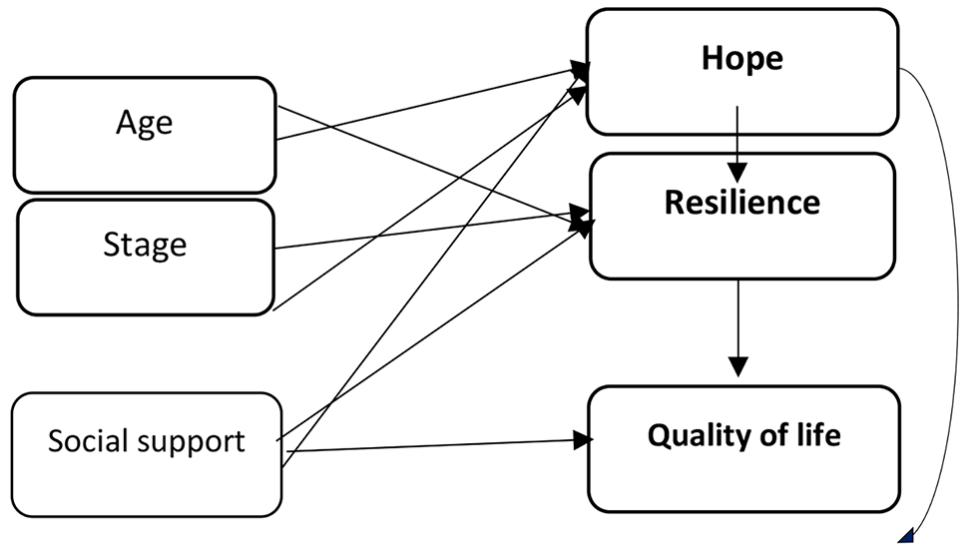
Conceptual Model

The conceptual model of the research is as follows (Fig. 1).

## Method

The present descriptive-analytical cross-sectional study has investigated the predictive factors of quality of life in women with breast cancer referring to the oncology Center of Valiasr International Hospital in Tabriz, Iran from May to December 2022. Women older than 18 years of age, with diagnosed breast cancer, without a history of other cancers, with complete knowledge of their disease and the ability to read and write were included in the study, and those who did not fill out the questionnaires completely or were not inclined to participate in the study, and those with concomitant diseases or serious medical conditions were excluded from the study.

### Sample size and sampling

Taking the study objectives into consideration, the largest sample size obtained was selected as the final sample size using the mean calculation formula. Considering the power of 90%, the confidence interval of 95%, and the error rate of 5% around the mean quality of life (Mean = 58.79, SD = 21.05) reported by Li et al., [24] the sample size of 198 people were obtained. Finally, considering the increased accuracy of the study, the sample size was added by 10%, and the final sample size was calculated to be 218 people. After obtaining the required permits, sampling was performed using the convenience sampling method until reaching the calculated sample size.

$$n=\frac{{({Z}_{1}-\frac{\alpha }{2})}^{2} \times {s}^{2}}{{d}^{2}}$$

### Study variables and measurements

#### 1- Baseline information

It includes the demographic variables of the people under study. The first part includes age, level of education, employment, marital status, self-assessment of the economic status of the family, and the disease characteristics section, including the duration of the disease and Stage of breast cancer.

#### 2- Health-related quality of life (SF-12)

Ware, et al., designed the Short-Form Health Survey (SF-12) [38], it is a shortened form of the SF-36 Health Survey (SF-36) [39], is a largely used instrument for assessing patient-reported general health conditions/ HRQoL. The instrument is categorized into eight health domains to evaluate physical and mental health, each including six items. Physical health scales include general health (1 item), physical functioning (2 items), role physical (2 items), and bodily pain (1 item). Mental health domains include vitality (1 item), social functioning (1 item), role emotional (2 items), and mental health (2 items). Scores for items range from 1 to 6. To enable comparison of the study results in different cultures, we used US population-derived SF-12 norms which consider a mean value of 50 and a standard deviation value of 10 [40]. Scores on this questionnaire are in the range of 0–100, where higher scores indicate a better self-perceived health status. The validity and reliability of this questionnaire in Iran have been evaluated by Montazeri et al. [41].

#### 3- Connor–Davidson Resilience Scale (CD-RISC)

The scale was by Connor and Davidson developed in 2003 [42]. t is consisted 25-item which to measure psychological resilience. Responses are indicated on a 5-point Likert-type scale, ranging from 0 (not true at all) to 4 (true nearly all the time). Items are divided into five subscales: (1) reflects the notion of personal competence, high standards, and tenacity, (2) corresponds to trust in one’s instincts, tolerance of negative affect, and strengthening effects of stress. (3) Relates to the positive acceptance of change and secure relationships, (4) is related to control, and (5) to spiritual influences. The total score ranges from 0 to 100, with a higher score reflecting higher resilience [42]. The Persian CD-RISC also has high reliability (Cronbach’s α = 0.91) and satisfactory validity [43]. In this study, Cronbach’s α values were 0.86.

#### 4- Hope Scale

designed by Snyder, et al., 1991 [44] and it is a 12-item Likert-type scale with four items assessing pathways, four items assessing agency, and four distracters. The HS yields separate scores for the Pathways and Agency Subscales, or the entire Hope Scale can yield one score. In this respect, confirmatory factor analyses across multiple college student samples support using the agency and pathways subscale in creating a higher-order hope factor. Response options range from 1 = false to 8 = true. Both Cronbach alphas (from 0.74 to 0.84) and test/retest reliabilities (0.73–0.82 over an 8–10-week period) are acceptable for the eight items in the two hope subscales [44]. The validity of the Hope Scale in Iran was investigated [45, 46].

#### 5- Multidimensional Perceived Social Support Scale Questionnaire

Perceived Multidimensional Social Support Scale by Zemit et al. [47] was prepared in 1998 to assess perceived social support from three sources of family, friends, and important people in life, with a minimum score of 12 and a maximum score of 84. A score of 12.48 showed low social support and a score of 49–68 showed a moderate level of social support and a score of 69–84 showed a high level of social support [47, 48]; its validity and reliability in Iran through content analysis and Cronbach’s alpha coefficient ranged from 86 to 90 It was calculated for the subscales and 86% for the whole instrument [48].

### Procedure

After coordination with the authorities of the hospital, as the most equipped private hospital in the northwest of Iran and a treatment center for cancer patients, sampling was performed based on the inclusion criteria. The researcher went to this hospital, and after identifying the patients based on the inclusion criteria, explaining the study objectives to them, and obtaining informed consent, the questionnaires were provided to the patients to fill out in a self-administered form.

### Data analysis

SPSS version 24 software and Lisrel 8.8 software were used for data analysis. Descriptive statistics were used to describe the participants’ demographic characteristics, and independent t-tests and ANOVA tests were used to assess the relationship between the variables of demographic variables and quality of life. Pearson correlation analysis was performed to assess the relationship of three independent variables, including resilience, social support, and hope, with the dependent variable of quality of life. In this study, we first, designed the conceptual model of the research and then the fitting of the model paths was evaluated. Model fit measures were obtained to conclude how well the suggested model caught the covariance among all the measures. The normal distribution of data was assessed using the Kolmogorov-Smirnov test, skewness, and kurtosis. Although the data were slightly skewed, the data were considered normal because skewness and kurtosis were 1 to -1.

## Results

### Sample characteristics and their relationship with quality of life

The participants were 218 women with breast cancer [mean age = 41.44 years, SD = 10.29, range = 20–61] and most of them were in their forties. 177 (81.2%) cases were married, and a total of 162 participants (74.3%) had less than one year since the diagnosis of their disease. In terms of breast cancer staging, the majority of women were in the second stage (n = 128, 58.7%). Other demographic information is presented in Table 1. The primary analysis of the relationship between demographic characteristics and quality of life demonstrated that the mean score of quality of life was significantly higher in housewives than in working women (48.54 vs. 43.60) (t = 2.119, P = 0.035). The levels of quality of life relative to income status (F = 3.097, P = 0.047) and the duration of disease diagnosis (F = 4.069, P = 0.018) were also significantly different in the participants so that the mean score of quality of life in individuals with a better income status was more than that in the groups with an unfavorable income status. This was also the case in individuals whose disease was diagnosed less than one year ago compared to those whose disease was diagnosed more than two years ago. It is worth noting that the mean score of quality of life was lower in women over 60 years old than in other age groups, and divorced and single women than in married women, and in women with elementary education than in those with higher education, but these differences were not statistically significant (Table 1).

**Table 1:** Participants’ demographic and disease characteristics and Relationship with quality of life (N = 218)

Variable	N (%)	Mean(SD)	t or F	P-value
**women’s age groups (year)**			F = 1.763	0.138
20–29	31 (14.2)	47.22(9.56)		
30–39	55 (25.2)	46.11(10.31)		
40–49	75 (34.4)	47.96(10.29)		
50–59	51 (23.4)	51.25(11.49)		
≥ 60	6 (2.8)	45.10(10.59)		
**women’s educational status**			F = 2.370	0.096
Primary and secondary school	65 (29.8)	45.90(11.17)		
Diploma	87 (39.9)	48.42(9.44)		
University	67 (30.7)	49.83(11.18)		
**women’s employment status**			t = 2.119	0.035
Housewife	171(79.5)	48.54(10.94)		
Employed	44(20.5)	43.60(6.51)		
**Marital status**			F = 0.298	0.743
married	177 (81.2)	48.27(10.41)		
single	29 (13.3)	47.99(12.50)		
Divorced	12 (5.5)	45.83(8.74)		
**Income status**			F = 3.097	0.047
More than enough	52 (23.9)	49.36(9.52)		
enough	118 (54.1)	48.89(10.91)		
Less than enough	48 (22.0)	44.79(10.43)		
**Stage of breast cancer**			F = 0.039	0.962
I	61(28.00)	47.81(10.97)		
II	128(58.7)	48.26(10.85)		
III	29(13.3)	47.99 (8.81)		
**Time since diagnosis**			F = 4.069	0.018
< 1year	162(74.3)	47.89(10.28)		
Between 1 and 2 years	44(20.2)	50.76(10.80)		
Between 2 and 5 years	12(5.5)	41.20(11.41)		

### Descriptive and correlation analysis of resilience, hope, and social support, with QOL

Baseline descriptive statistics for resilience, life expectancy, perceived social support, and quality of life are represented in Table 2. The mean score of resilience was 59.22 (SD = 17.25, range = 12–100), the mean score of hope was 38.53 (SD = 6.19, range = 33–53), and the lowest score was related to the pathway thinking subscale or planning for goal achievement. Also, the mean score of social support was 59.37 (SD = 16.68, range = 12–86), the score of perceived support by others was higher than that of other areas, and the support received from friends obtained the lowest score. Finally, the mean score of quality of life was 48.10 (SD = 10.59, range = 19–75), and the score of the physical domain was lower than that of the mental domain. The results of the Pearson correlation test between the main variables of the study are presented in Table 2. The relationship between resilience and total social support was shown to be significant, positive, and strong (r = 0.684, P < 0.001). Also, the results indicated that the relationships of total social support, resilience, and hope with the quality of life of women with breast cancer were positive and moderate; however, the correlation between the two variables of perceived social support and quality of life was higher.

**Table 2:** Descriptive and Correlations between the main Variables of the Study

Variable	Mean (SD)	Min	Max	1	2	3	4
1-Total CD-RISC	59.22(17.25)	12	100	1	0.684*	0.443*	0.474*
tenacity	19.12(6.25)	3	32				
tolerance of negative affect	11.22(4.33)	3	20				
positive acceptance of change	14.57(5.60)	3	28				
control	7.36(2.57)	0	12				
spiritual	6.61(1.42)	0	8				
2-Total MSPSS	59.37(16.68)	12	86		1	0.481	0.533*
Social support from specific people	21.06(5.28)	4	28				
Social support from Friend	17.29(6.23)	4	28				
Social support from Family	20.48(6.78)	4	30				
3-Total Hop (AHS)	38.53(6.19)	13	53			1	0.411*
agency	12.76(2.88)	4	20				
pathway	11.83(2.71)	4	18				
4-Total SF-12 score	48.10(10.59)	19	75				1
Physical component score	12.01(2.59)	7	19				
Mental component score	17.30 (2.44)	10	26				

^*^P < 0.001

**Fig. 2:**
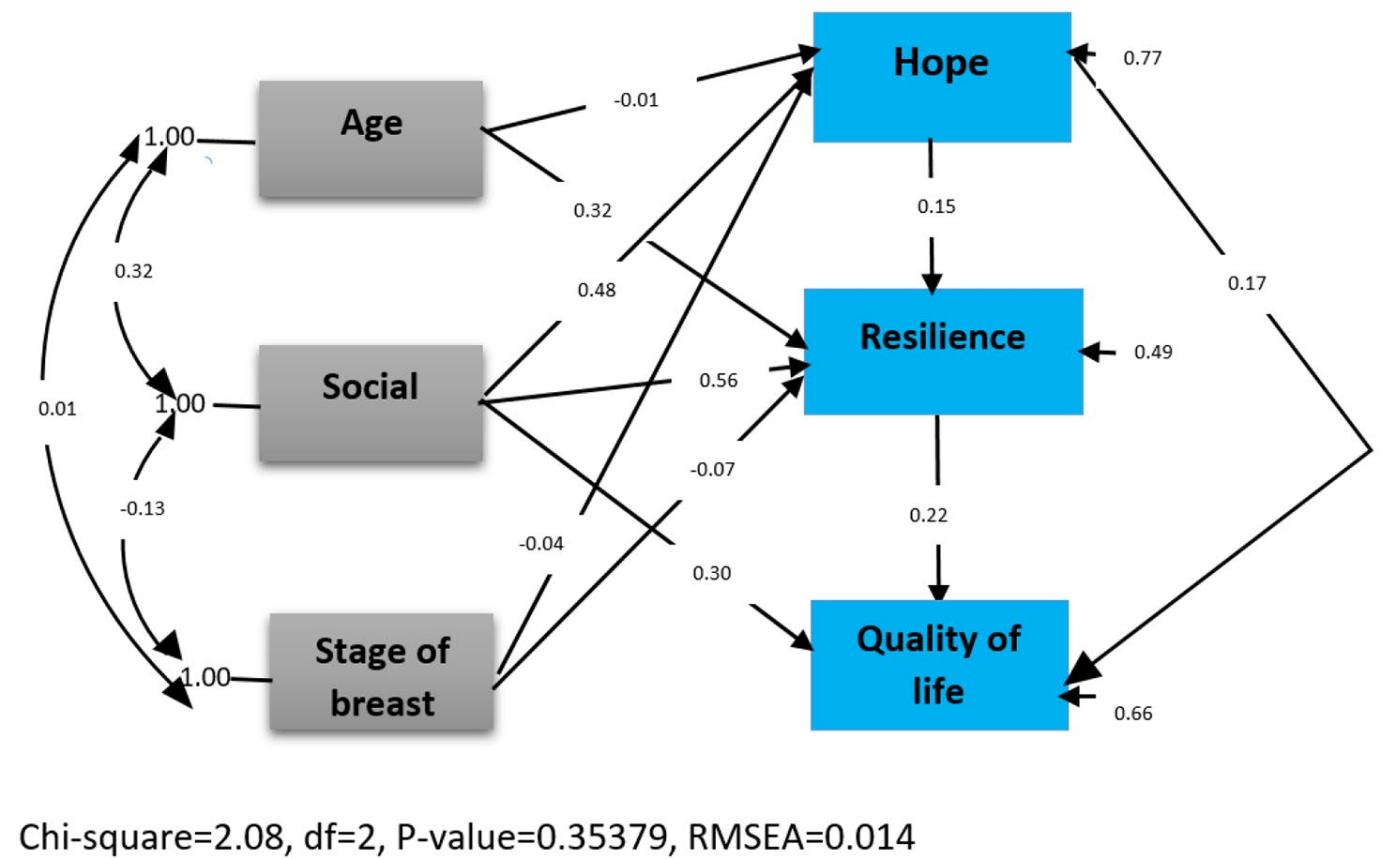
Path diagram of the relationship of Health-related quality of life and five predictors. Values represent standardized regression coefficients

Furthermore, Fig. 2 depicts the conceptual model assessed by pathway analysis. Based on this analysis, the fit indices of the HRQoL prediction model were as follows.

[Normed Chi^2^ = 2.08 < 5.0, RMSEA = 0.014 < 0.060, GFI = 0.99, both CFI = 0.99 and IFI = 1 > 0.90)].

The data support the conceptual model, and the indices denote a good fit for the model. If the Chi^2^ /df ratio was obtained equal to 1.04 and between 1 and 2, and p-value = 0.353, and the root mean square error of approximation (RMSEA) index was obtained less than 0.06, based on the pathway analysis, the relationship between age and hope was negative, but not significant (T-value=-0.14). Also, although a negative relationship was obtained between the disease stage variable and the two variables of hope and resilience, these relationships were not significant either, so the T-value was − 0.74 and − 1.34, respectively. Other variables were significantly related (T-value > 1.96).

Based on Fig. 2 and the results of Table 3, the independent variables of resilience, life expectancy, and perceived social support not only directly affected the HRQoL, but the two variables of hope and perceived social support also indirectly affected the quality of life variable. All three independent variables could explain 34% of the changes in the quality of life variable (adjusted R^2^ = 0.34). Furthermore, the variables of age, disease stage, and social support could explain 23% of the changes in the hope variable (adjusted R^2^ = 0.23), and the four variables of age, disease stage, life expectancy, and social support could explain 51% of the changes in the resilience variable (adjusted R^2^ = 0.51). In addition, as shown in Table 3, the age variable had an indirect effect on the dependent variable of quality of life through the resilience variable, and perceived social support showed the highest total effect on the quality of life variable.

**Table 3:** Standardized effects of study variables on the health-related quality of life in a woman with breast cancer (n = 218)

Variable	Direct effects	Indirect effects	Total effects
Hope	0.17	0.033	0.203
resilience	0.22		
Social support	0.30	0.4152	0.7152
Age		0.031	0.031
Stage			

## Discussion

The main objective of the study was to assess the effects of factors on the quality of life and their direct and indirect effects on the quality of life of women with breast cancer. The selection of the main predictors of quality of life, including social support, resilience, and life expectancy, and other factors, including age and disease stage, was based on the findings of previous studies. The conceptual model was evaluated by the pathway analysis, and the results indicated that the conceptual model had a good fit for the data, and the results supported the research hypothesis. The findings revealed that HRQoL was reasonably predicted by social support, life expectancy, resilience, and factors such as age and was not influenced by disease stage so that the independent variables had positive or negative effects on each other, as well as direct and/or indirect effects on the patient’s quality of life.

First, the primary investigation obtained from this study showed that the women with breast cancer participating in the study suffered from poor and impaired quality of life because the total mean score of quality of life was lower than that reported by patients with breast cancer in other studies. In studies conducted in other countries, patients with breast cancer obtained relatively higher scores in the total quality of life [12, 49]. Moreover, in our study, the score of the physical health domain was lower than that of mental health. In general, the quality of life in afflicted women has not been reported at an appropriate level, so in other studies, the score of quality of life in patients with breast cancer was also lower than that in the general population [17, 50]. An inappropriate quality of life can be affected by factors such as patients’ socio-demographic characteristics, including religion, and the characteristics of the disease itself. Also, the scores obtained for social support (59.37 vs. 66.08), hope (59.37 vs. 47.66), and resilience (59.37 vs. 60.36) are lower than those in the general population of women reported in Iran. In other studies, also, individuals with cancer scored lower than the general population in the mentioned items [51], requiring the importance of paying attention to this issue and implementing related interventions.

Second, this study investigated the role of social support in the relationship among resilience, hope, and quality of life in breast cancer patients. The results indicated that all three independent variables directly and significantly affected HRQoL, and social support not only had a direct effect and a moderate relationship with quality of life, but the path analysis also showed that quality of life could indirectly affect patients through hope and resilience. These results reveal the importance of social support about improving the patient’s quality of life, a finding that corresponds to other available evidence that supports the helpful effect of social support [14, 52, 53]. Social support can impact the adjustment process and HRQoL in several ways. The emotional support received from family, friends, and healthcare providers may help patients in the physical recovery process or create the ability to cope with the challenges of the disease and related treatments, therefore, allowing them to have a good quality of life so that the obtained support can be reassuring in the process of cancer diagnosis and treatment, and by creating a sense of value in the patients in the difficult situation they are experiencing, it can minimize the disturbing effects due to the disease.

Furthermore, the findings indicated that hope and resilience had poor relationships with quality of life. However, the pathway analysis is based on the role of social support and its effect on these two variables in such a way that social support with a strong and moderate relationship with resilience and hope, respectively, has been able to considerably affect the total quality of life. In this regard, in a qualitative study on cancer patients, the patients described the received social support as a facilitating factor for hope, which can impact quality of life [54]. In another study on elderly women of the general population, the importance of social support in maintaining hope has been underlined [55]. Also, providing interventions in support groups among cancer patients has been mentioned as a source for promoting hope [56]. Other studies also support hope as a mediator between social support and psychological consequences, confirming the results of the present study. Concerning resilience and its effect on quality of life, the literature review indicates that patients’ high resilience can positively affect the health outcomes of patients [14, 57]. In a recent study by Liang and colleagues, it was found that, when compared to other risk factors, resilience emerges as a superior predictor of decreased quality of life in the following year [58]. Researchers also reported that social support was a stress-modulating factor and an effective result on health outcomes on the one hand [59, 60]. On the other hand, it is probably a factor facilitating the activation of coping mechanisms that are helpful in stressful situations [61, 62]. Therefore, breast cancer patients with higher resilience may use an active coping style influenced by social support to improve their current quality of life. Recently, Dewi et al. reported using a coping strategy has a partial or simultaneous positive impact on the quality of life of breast cancer patients [63]. According to the results of studies conducted on animals, social support may inhibit the hypothalamus-pituitary-adrenal (HPA) axis reaction to stress [64–66], and resilience to stress is associated with the regulation of noradrenergic activity produced by the HPA system in an optimal window [67]. By multiple regression analysis, Filazoglu and Griva [17] showed that disease-related variables such as cancer stage and psychological factors such as social support and problem-solving coping were considerable predictors for HRQoL. The independent variables of hope and resilience also directly and significantly affected HRQoL.

Finally, based on the developed conceptual model, the age variable positively and negatively affected resilience and hope, respectively; however, it was significantly related only to the resilience variable, and the relationship between the two variables of age and hope was not statistically significant, and the age variable showed only a 3% indirect effect on the quality of life via its effect on resilience. Regarding the disease stage variable, although it was negatively related to the two variables of resilience and hope, i.e., by increasing the disease stage, resilience, and hope scores decrease, this relationship was not statistically significant. There is evidence that young women are resilient after a breast cancer diagnosis and have HRQoL the same as women who have breast cancer. Research shows that cancer patients at a young age, compared to older patients, not only have different experiences, but their response to the disease is also different; for instance, different age groups have different ways of dealing with the disease. Researchers have found that older patients consider social support from healthcare providers important, while younger patients consider expressing their feelings more important. These differences can be due to the presence of various challenges in each age group when dealing with cancer; for example, young patients have encountered the concern of caring for their older parents or the effect of the disease on their children, while older people are more concerned about their own physical and cognitive restrictions. In a systematic review [36], researchers reported that no relationship was found in 9 studies between age and hope. However, one study revealed that the total score of hope was inversely associated with age. Moreover, in this systematic review, it has been shown that in 3 studies, no relationship was found between hope and different stages of the disease. However, in Hasson-Ohayon et al.’s study [68], a negative relationship was reported between the disease degree and hope. Concerning resilience, Cohen et al. [69] reported that older people possessed higher resilience, but no relationship was found between disease stage and resilience, which is consistent with our study.

### Strengths and limitation of the study

We designed a model for assessing the effects of psychological/behavioral parameters on health-related quality of life among women with breast cancer. We found not only the direct effects of predictor variables on the health-related quality of life but also we found their indirect and total effects.

The limitations of the current study include the following. First, as it is obvious, studies with a cross-sectional design cannot provide a more detailed explanation of the causal relations between variables. The heterogeneous population characteristics of the present research may have caused bias in our results. Further studies can also be designed with a larger sample size and specific to different groups. Besides, in our study, resilience and hope were regarded as mediating variables to assess the relationship between social support and quality of life, while other factors, such as self-efficacy, optimism, ability to cope, etc., may also affect the relationships between variables in individuals with breast cancer, which requires the examination of other variables. In this regard, qualitative studies can also be considered to explain the meaning of relationships between factors contributing to the quality of life. This study had some strong points such as.

## Conclusion

Our study patients had relatively moderate HRQoL, resilience, and life expectancy; however, this study indicates the importance of social support in HRQoL in breast cancer. The present study showed that psychosocial factors and age are independent predictors of HRQoL, and taking psychological and demographic characteristics into account during the diagnosis and treatment of women with breast cancer is of particular importance, and healthcare professionals must be familiar with the factors involved in the quality of life of breast cancer patients and adopt selected strategies and interventions based on each person’s characteristics and disease. Finely, these findings serve as a reminder to oncologists and healthcare workers to consider the concept of hope, resilience, and the impact of social support on these concepts, and by examining these factors and considering the characteristics of each patient such as age-related changes, perform their supportive and therapeutic measures. And ensure that the patients’ mental needs and aspirations align with the proposed treatments.

## Data Availability

The datasets are available from the corresponding authors on request.

## Abbreviations

HRQoL: Health Related Quality of Life
CD-RISC: Connor–Davidson Resilience Scale
MSPSS: Multidimensional Scale of Perceived Social Support
RMSEA: Root Mean Square Error of Approximation
